# Vanishing Point Detection Method Based on Constrained Classification for Checkpoints on Urban Roads

**DOI:** 10.3389/fbioe.2022.920329

**Published:** 2022-07-04

**Authors:** Guoqiang Gong, Junqing Liu, Zhengxiao Li

**Affiliations:** ^1^ College of Computer and Information, China Three Gorges University, Yichang, China; ^2^ International School, Beijing University of Posts and Telecommunications, Beijing, China

**Keywords:** vanishing point, line detection, CHT, checkpoints on urban roads, line segment classification

## Abstract

To solve the problems of computational complexity and inaccuracy in classical vanishing point detection algorithms, such as the cascaded Hough transform, a vanishing point detection method based on constrained classification is proposed. First, the short line data are filtered to avoid interference in straight line detection, and then, the line segment is screened and classified by hierarchical clustering according to the image characteristics of the line segment and the variation pattern of angle similarity. Subsequently, Three types of straight line segments with the most significant angle differences are acquired. To prevent the optimization algorithm from getting stuck in the “wrong” local optimum neighborhood or failing to locate the global optimum, a set of constraints are set to further restrict the search. Afterward, the classified line segments are projected into a finite rhombic space, which are then quantified. The point with the maximum vote is eventually identified as the vanishing point. Experimental results show that the proposed method not only greatly reduces the computational complexity of vanishing points but also largely improves the accuracy of vanishing point detection.

## Introduction

Vanishing point is an important feature found in most images in real life and has a wide range of applications, such as camera positioning and calibration (Lee and Park, 2020; Tang et al., 2019), as well as scene reconstruction (Li F. et al., 2019). With the continuous construction of smart cities, a huge number of surveillance cameras have been distributed at checkpoints on urban roads mainly for taking snapshots of cars driving through a red light, as speed traps, for traffic security management, etc. Effective calibration of checkpoint cameras is the key to better implementing these functions or utilizing the cameras for more complicated scenarios, such as line marking violation detections and vehicle tracking. Cameras may be calibrated manually, semiautomatically, or automatically, and specifically for the automatic camera calibration, automatic detection of vanishing points is the main premise for realizing its functions.

At present, there are many excellent methods for detecting vanishing points, such as random sample consensus (RANSAC) (Bazin and Pollefeys, 2012; Li, 2009; Mirzaei and Roumeliotis, 2011), in which vanishing points are calculated via continuous iteration. However, most of these methods cannot ensure the optimization of their solutions, which leads to poor robustness. There is another method based on a texture algorithm (Bui et al., 2014; Wu et al., 2016). In this method, the vanishing points in unstructured and structured roads can be accurately detected by searching the main direction of the texture in the image, and then, the voting result can be used to locate the vanishing points. Nevertheless, this method entails a high computing cost and poor timeliness. Currently, the Hough transform, which employs an edge-based algorithm, is a method that shows a relatively excellent performance (Liu and Zhou, 2015; She et al., 2015). It extracts straight lines in an image using an edge detector for the Hough transform and uses voting to locate vanishing points. This method has a high computation speed, but it depends on the strong edge in the image. Therefore, it is more suitable when applied to structured road images. This method has been continually improved and perfected. A cascaded Hough transform (CHT) based on parallel coordinates (Dubska et al., 2011; Markéta and Herout, 2013) was used to map a real projective plane to a finite rhombic space, extending the application scope of vanishing point detection. However, the major problem is that the line data for the Hough transform are messy, resulting in a complicated and inaccurate calculation. There are some other methods of object detection algorithm by deep learning (Jiang et al., 2021a; Jiang et al., 2021b) that can restore the vanishing point (Bai et al., 2022; Hao et al., 2022); however, they are certainly complex (Joo et al., 2019), which are supported by computing resources (Li G. et al., 2019).

To solve this problem, a method for detecting vanishing points based on constrained classification was proposed for the intelligent application on an image captured from a checkpoint on an urban road. The core idea revolves around reducing the number of initial straight line segments fed to the Hough transform through screening and classification, thereby lowering the computational complexity of vanishing points, reducing the interference data, and ultimately improving the accuracy of vanishing point calculation.

## Vanishing Point Detection Method at Checkpoints on Urban Roads

**FIGURE 1:**
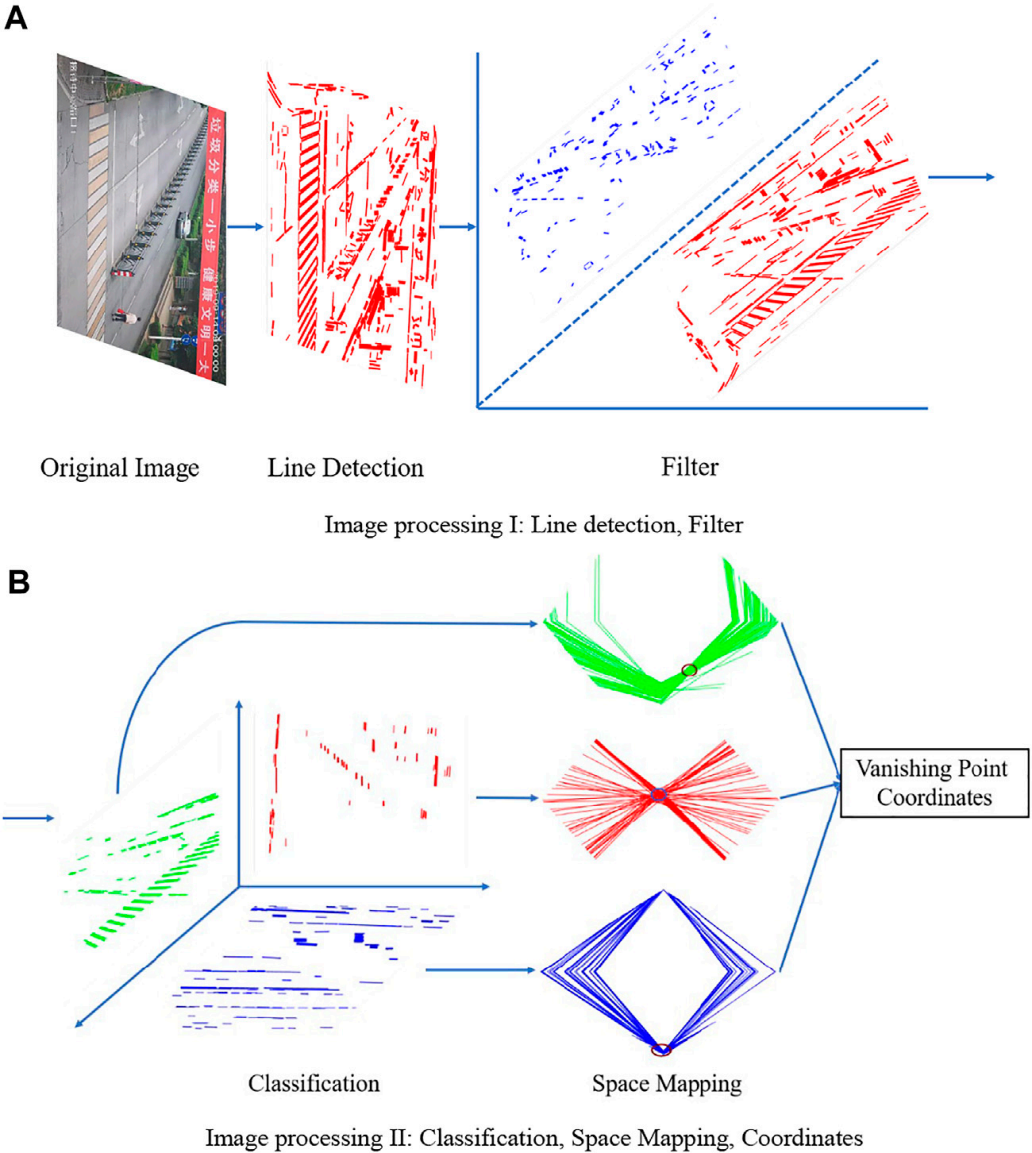
Flowchart of the proposed vanishing point detection method. **(A)** Image processing I: line detection, filter. **(B)** Image processing II: classification, space mapping, coordinates.

This paper mainly discusses the detection of vanishing points in surveillance camera images captured at checkpoints on urban roads, which were assumed to be basically straight. The vanishing point detection method is mainly divided into three modules. Module 1 is used for straight line segment detection based on the LSD algorithm and filters the line segments. Module 2 is used for the screening and classification of straight line segments and classifies line segments into three categories according to the significant features of images at the checkpoints and the variation pattern of angles made by straight line segments. Module 3 is used for the CHT based on parallel coordinates and maps the line segments obtained from Module 2 to a rhombic space, respectively, to facilitate the use of correlation calculation to obtain the coordinates of the vanishing point in the image. Figure 1 shows the method process.

### Line Detection and Filtering Method

LSD is a line segment detection and segmentation algorithm (Flexa et al., 2019). A large number of short lines would be generated during the detection because of the self-growth feature of the local detection algorithm and the complexity of the environment at the checkpoint, which increases the complexity of the subsequent vanishing point solution. Moreover, the direction angle of the short lines is extremely sensitive to the straight line endpoint coordinate. A large number of short lines will constitute a strong noise in the subsequent processing, indirectly causing the straight line detection method to greatly limit the subsequent vanishing point calculation. In this paper, short line segments among the straight line segments from the LSD algorithm were filtered to improve the speed and accuracy of vanishing point detection. The method will be described in greater depth below.

When the LSD algorithm detects a line, the length *L* of each line segment is calculated from the coordinates of two endpoints. The vector direction was set to be the direction at the endpoint of a straight line segment with a large *x*-axis value. The angle (0°–180°) between the vector $\vec{X}$ and the *y*-axis direction vector $\vec{Y}$ in the image coordinate system is calculated using the following formula. The angle data of the straight line is denoted as *α*. The length *L* of the straight line segment has a one-to-one correspondence with angle *α*.

$$\alpha =arccos\frac{\vec{X}\cdot \vec{Y}}{\left|\vec{X}\right|\cdot \left|\vec{Y}\right|}$$ (1)

**FIGURE 2:**
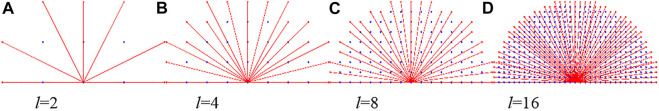
Relationship between segment length *l* and angle resolution.

Note that the line segment coordinates required for calculating the straight line angle are pixel coordinates. Therefore, for digital images, the segment length is a discrete value in the interval of positive real numbers. Besides, for images with a given pixel resolution, the segment length is a finite number of discrete positive real numbers. The segment length takes a finite number of discrete values, which means that the angle between the lines is also finite, rather than any value from 0° to 180°. Figure 2 shows the angle between the straight line and the coordinates when the segment length *l* = 2, 4, 8, and 16, such as Figures 2A–D (the image coordinates are rounded after calculation).

In Figure 2, red and blue points represent pixels, whereas the red lines are possible lines with a given length, for example, in Figure 2A, when the line length is 2 and the possible values of the angle between the line and the coordinates are 0, 30, 60, 90, 120, 150, and 180, there are seven lines with a length of 2. In such a case, the minimum angle resolution is 30°. That is, when the line coordinate moves a pixel, the angle between the lines changes by 30°. Similarly, in Figure 2D when the segment length *l* = 16, there are only 57 values of the angle between the line segment and the coordinates. In such a case, the minimum angle resolution is 3.21°. Table 1 shows the conditions for segment length *l* between 1 and 30, where *m* represents the number of line segments and *Δ* represents the angle resolution, i.e., the average degree of change in angle corresponding to a pixel change in line coordinates.

**TABLE 1:** Relation between segment length and angle resolution.

*L*	1	2	3	4	5	6	7	8	9	10
*m*	5	7	9	17	15	21	21	25	35	29
*Δ*	45.00	30.00	22.50	11.25	12.86	9.00	9.00	7.5	5.29	6.43
*L*	11	12	13	14	15	16	17	18	19	20
*m*	37	35	45	45	43	57	57	57	59	57
*Δ*	5.00	5.29	4.09	4.09	4.29	3.21	3.21	3.21	3.10	3.21
*L*	21	22	23	24	25	26	27	28	29	30
*m*	73	71	73	73	85	83	81	93	87	101
*Δ*	2.5	2.57	2.50	2.50	2.14	2.20	2.25	1.96	2.09	1.80

As shown in Table 1, when the segment length is 20, the angle resolution is 3.21°. For an image with a resolution of 720 × 480, a pixel change will cause an angle change of 3.21°, which is too demanding for the accuracy of line detection. For existing straight line detection methods, the detected line segments whose lengths are below 20 cannot be used as a basis for certain judgments. This implies that the angle between short lines has great randomness. In this paper, to verify this conclusion, the results of LSD line detection were statistically analyzed as follows.

First, the concept of clutter on the straight line was introduced to describe the degree of cluttering in the angular distribution of lines. Clutter reflects the degree of angular changes occurring to the line, shown in the formula below. In the formula, *N* is the number of lines within the length interval, *α*_*i*_ (*i* = 1,2, …,*N*) is the angle of lines with the corresponding length, and *μ* is the mathematical expectation of the angle of lines.

$$M=\frac{\sum_{\text{i}=1}^{\text{n}}{\left(\alpha_{\text{i}}-\text{μ}\right)}^{2}}{\text{n}}$$ (2)

**FIGURE 3:**
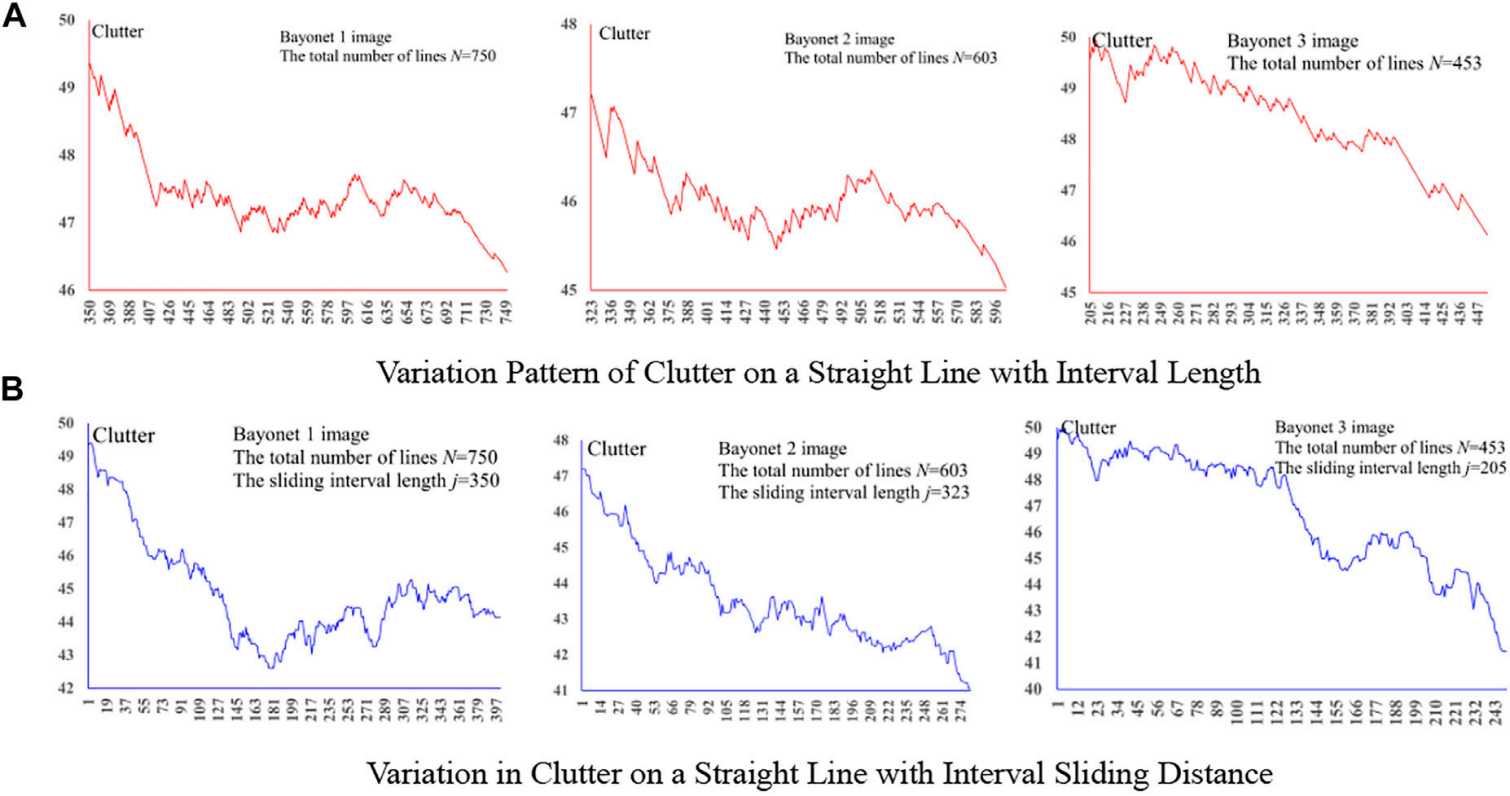
Variation pattern of clutter on the straight line. **(A)** Variation pattern of clutter on a straight line with interval length. **(B)** Variation in clutter on a straight line with interval sliding distance.

Several images captured at various checkpoints on urban roads were randomly selected. All lines detected in each image were sorted into the length *l*_*i*_ (*i* = 1,2, …,*N*) to obtain the corresponding sequence of angles *α*_*i*_ and form an angle set *S*_*N*_ = {*α*_*i*_|*i* = 1,2, …,*N*}. Angle *α*_*j*_ (1 < *j* < *N*) was closest to 20°. The clutters of the following angle intervals *S*_*M*_ = {*α*_*x*_|*x* = 1, …,*M*, *M* = *j*, …,*N*} were calculated successively. The variation curve for the clutters, as shown in Figure 3A, was also drawn. Meanwhile, the clutter within the sliding interval *S*_*y*_ = {*α*_*y*_|*y* = *n*, …,*j* + *n*-1,*n* = 1, …,*N* − *j* + 1} was calculated successively. The clutter curve drawn is as shown in Figure 3B. According to Figure 3A, as the angle interval *S*_*M*_→*S*_*N*_, the clutter decreased as a whole, indicating that the angle distribution pattern becomes more and more obvious with the increase in line length. The same conclusion can also be drawn from Figure 3B, wherein the clutter gradually decreases as the angle interval (fixed length) slides toward long lines.

In this paper, a total of 3,000 surveillance camera images with and without vehicles at 15 checkpoints in a certain region were selected for straight line detection and short line filtering. The resolution of all the images was 720 × 480. The line filtering threshold was set to 20. The following table lists the number of lines in the LSD detection as well as after the filtering and line reduction at some of these checkpoints.

**FIGURE 4:**
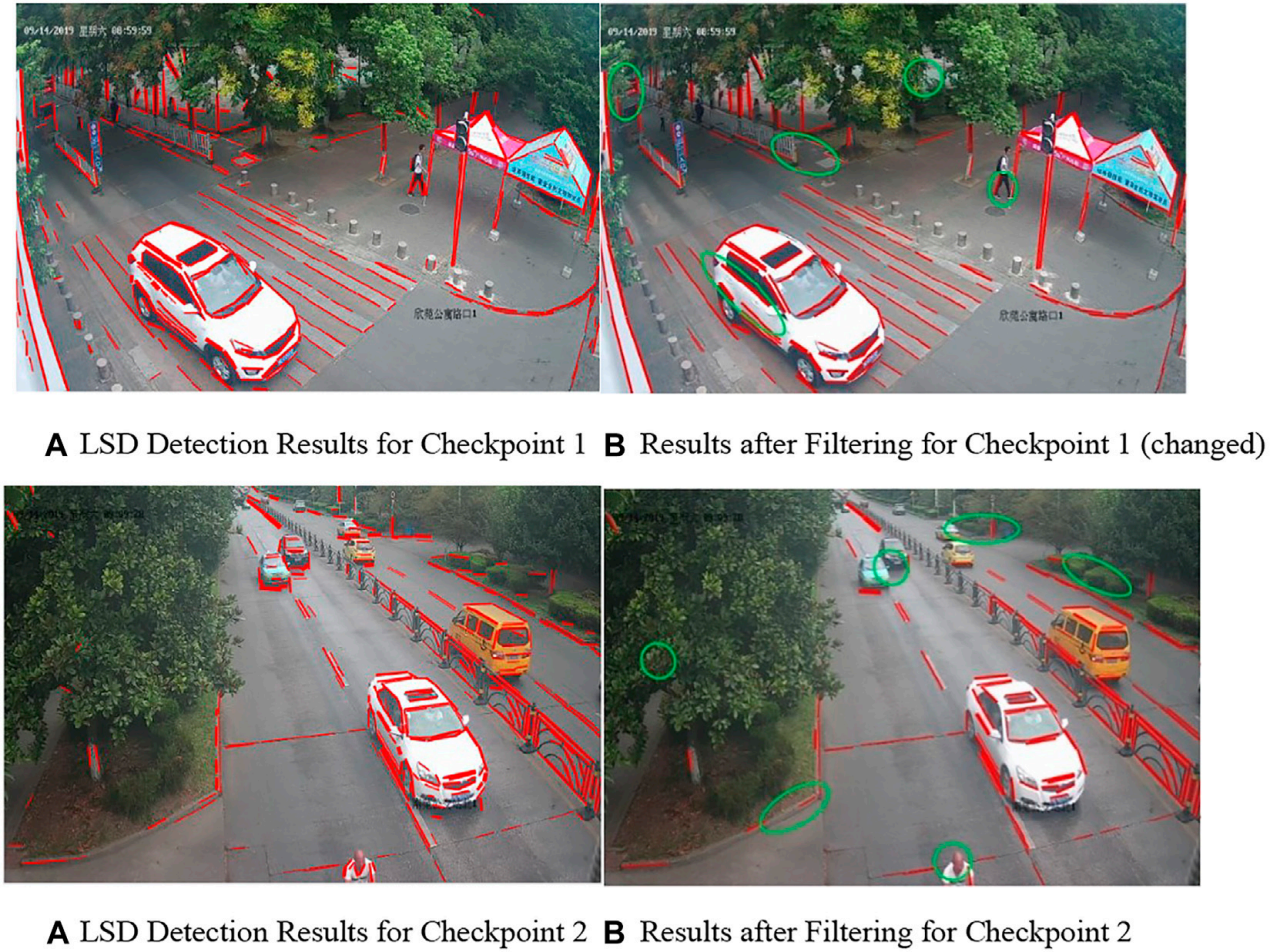
Results of line detection and filtering. **(A)** LSD detection results for checkpoint 1 **(B)** results after filtering for checkpoint 1 (changed). **(A)** LSD detection results for checkpoint 2 **(B)** results after filtering for checkpoint 2.

According to Table 2, the number of lines filtered out at the same checkpoint was very close no matter whether there are vehicles in the image. This also indicates that individual vehicles do not affect the overall dispersion distribution of the image. Since more lines were detected when there were vehicles, the reduction in such a case was slightly lower than that when there was no vehicle. Figure 4 shows the results of line detection performed via LSD and the filtering out of short lines in images at some checkpoints. The green ellipses in Figures 4B,D marked some of the short lines filtered out. In combination with the data in the table, it was possible to use the variation pattern of the clutter on the straight line to remove up to 20%–30% of short lines from all the straight lines detected using LSD, hence realizing effective filtering of short lines with irregular angular change and cluttering.

**TABLE 2:** Comparison of line detection results.

Image	Are there any vehicles?	Number of lines detected	Number of lines after filtering	Number of lines filtered out	Reduction (%)
Checkpoint 1	Yes	545	427	118	21.65
	No	423	318	105	24.82
Checkpoint 2	Yes	332	234	98	29.52
	No	230	146	84	36.52
Checkpoint 3	Yes	350	278	72	20.57
	No	284	216	68	23.94
Average of 15 checkpoints	Yes	462.6	351.5	111.1	24.35
	No	360.8	234.2	106.6	29.55

### Analysis of Line Angle (Direction)

**FIGURE 5:**
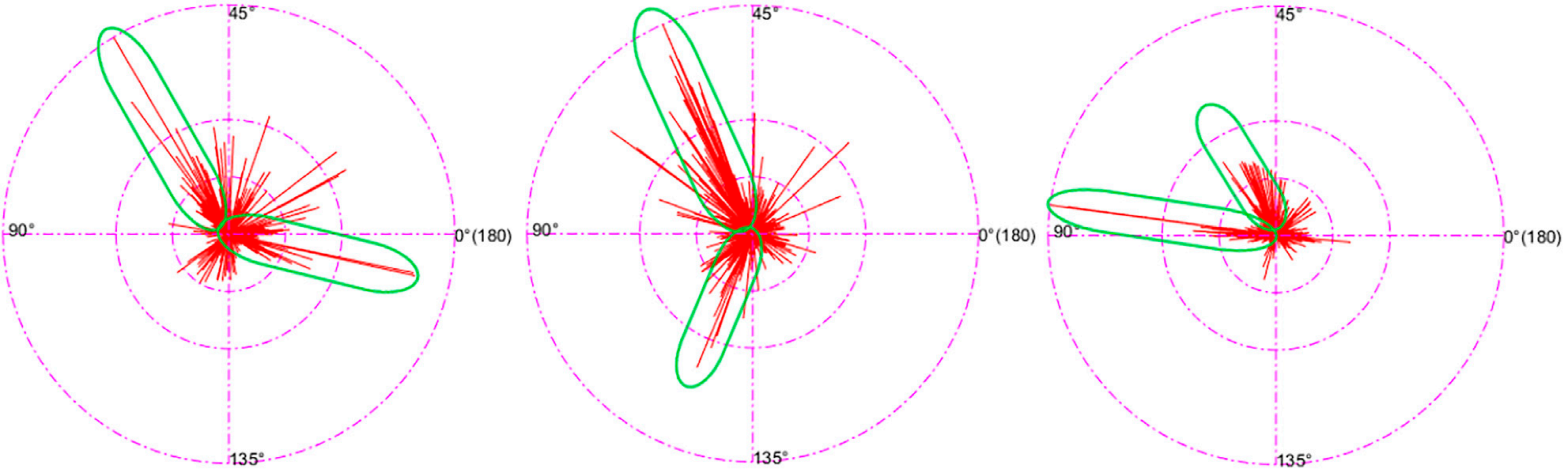
Line angle and length distribution diagram.

The images at checkpoints on urban roads have some significant features because of the application scenario. These features will be analyzed using the following data. The length of the straight line was corresponded to its angle in this section and presented on a circular metric denoting 0°–180° going full circle. Figure 5 shows the specific distribution of lines in several checkpoint images. The parts with a concentrated distribution of long lines are circled. It can be seen that the long line segments generally converge toward a certain angle range.

The angle range was respectively divided into 18 and 9 angle intervals on average with the angle interval length of 10° and 20°. The lines within each interval under the above two kinds of classification were counted, and the proportions of the number of lines within the first two intervals having the most lines out of the total number of lines were calculated. Similarly, the proportions of the number of lines within the first three intervals and the first four intervals out of the total number of lines in a total of 30 images were calculated. The mean was also calculated, and Table 3 shows all the results.

**TABLE 3:** Proportion of the number of lines in different intervals.

Classification	The first 2 intervals	The first 3 intervals	The first 4 intervals
9 intervals	0.498	0.615	0.710
18 intervals	0.383	0.460	0.547

According to the data in the table, very few angle intervals account for the majority of lines. It can be held that the lines in this kind of image are mainly lines making up certain angles. The following conclusion can be drawn from the significant geometric features at the checkpoints and Figure 5. The lines in the images at the checkpoints can be mainly classified into three categories: the first category involves lines that are parallel to the road surface, such as lane lines, road boundaries, and the longitudinal edges of the vehicle; the second category is lines that are horizontally perpendicular to the first category, such as passage lines and transversal edges of the vehicle; and the third category is lines that are vertically perpendicular to the road surface, the most obvious examples are roadside lights, traffic lights, and guardrails. In consideration of how the snapshots were normally taken, no matter where the camera is specifically located, and regardless of the elevation and deflection angle of the camera, the general angles made by each of the three line categories are significantly different from one another, whereas the variation within the same category is very small. Three kinds of lines with the most number of lines and the most significant difference in the angle can be obtained by selecting and classifying the lines according to the geometric variation pattern of the angles made by the lines, among many other methods.

### Screening and Classification of Lines

To accurately obtain the sets for the three kinds of lines, the improved AGNES (Guha et al., 2000) algorithm, a hierarchical clustering algorithm employing a bottom-up aggregation strategy, was adopted. The preset number of clusters was finally reached by constantly looking for the nearest two clusters to merge. Here, the Euclidean distance was used to calculate the distance between *A* and *B* from a single data point (the initial cluster), and the mean distance (uniform connection algorithm) was used to calculate the distance between the combined data points (clusters) *C*_*i*_ and *C*_*j*_. The formulas are as follows:

$$dist=\sqrt{{\left(A-B\right)}^{2}}$$ (3)

$$d_{avg}=\frac{1}{\left|C_{i}\right|\left|C_{j}\right|}\sum_{x\in C_{i}}\sum_{y\in C_{j}}dist\left(x,y\right)$$ (4)

The data clustered in this paper were the angle data of the straight lines. When these data were sorted by the angle, without considering the serial numbers of the lines, the data were one-dimensional. Moreover, to ensure that the selected line sets have the maximum angular similarity, the screening of target lines should be performed in line with a continuous interval (successive sequence of serial numbers) to ensure that the selected line sets have the maximum angular similarity. Therefore, the steps for calculating the data distance were simplified during hierarchical clustering. Only the distances between the cluster and the two before and after this cluster were calculated when calculating the distance matrix *N*. In this way, the clusters must extend to the left and right sides adjacent to them when they are merged, thereby ensuring that the clusters are arranged in a continuous serial number interval and that the amount of calculation can be reduced to a certain extent.

Algorithm 1 Angular Similarity Clustering Algorithm.

**Input:** $Cluster\;D=\left\{x_{1},x_{2},\ldots ,x_{m}\right\};Distance\;measure\;function\;d$

**Output:** N (j,i).

1 for  j=1,2,…,m  do

2 $C_{j}=\left\{x_{j}\right\}$

3 end for

4 for i=2,3…,m−1 do

5 for j=i−1,i+1 do

6 $N\left(i,j\right)=d\left(C_{i},C_{j}\right)$

7 N(j,i)=N(i,j)

8 end for

9 end for

In the most ideal case, after performing hierarchical clustering on the angle data, three clusters with the largest sample size, which represent the three kinds of target lines, are to be obtained. However, in reality, some redundant lines will be inevitably generated because of the different image structures and line detection algorithms. Since such lines form angles that are quite different from the three kinds of target lines, a series of noises and outliers will be generated. In such a case, selecting the appropriate number of target clusters becomes the key. When too many clusters are selected, there is a high probability that a small part of target lines or error lines will be selected. However, when the number of clusters selected is too few, the results obtained tend to be inaccurate because of the proportionally large amount of noise. Given the shooting angle of images and videos, the lens must be perpendicular to the ground in the vertical direction in most cases. It can be assumed that the third line category mainly gathers within the 0°–10° and 170°–180° angle intervals, so the head cluster and tail cluster were selected as candidates from the clustering results. In combination with the other two line categories, the number of target clusters should be greater than or equal to 4. The selection condition for the number of clusters $K=\left\{4\cdot x|\left(x-1\right)x/2<M/100,\left(x+1\right)x/2\geq M/100,x\in {\text{N}}^{\text{*}}\right\}$ is given here according to the number of lines. In this formula, *K* is the number of target clusters and *M* is the number of lines in the image (after filtering).

**FIGURE 6:**
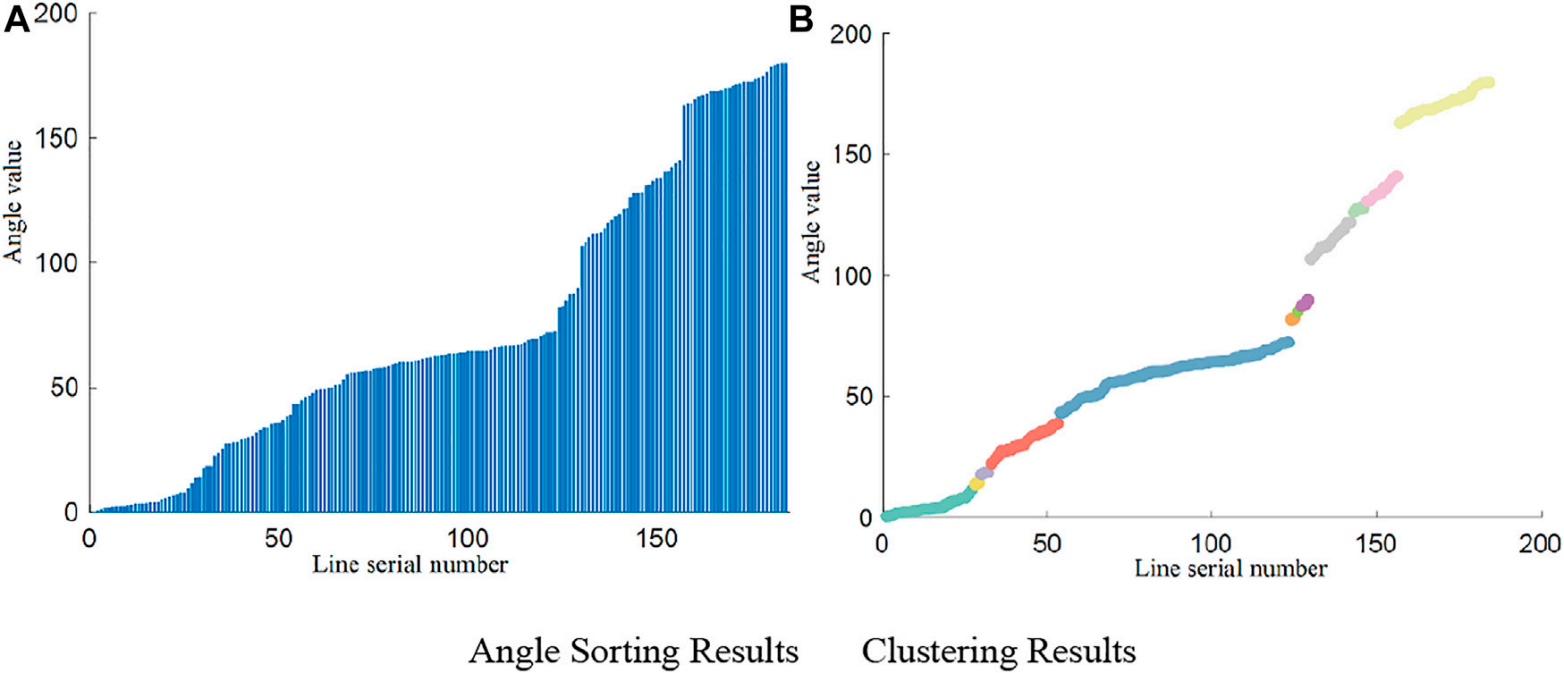
Sorting and clustering of angles. **(A)** Angle sorting results. **(B)** Clustering results.

Figure 6A shows the angle data sorted according to the angle sequence. The results of hierarchical clustering of the angle data are shown in Figure 6B, in which the curved areas in different colors represent different clusters. Clusters with the top five sample sizes, except for the head cluster and the tail cluster, were then selected from the images at several checkpoints, after which the mean of the angles and the proportion relative to the total number were calculated. The results are listed in Table 4 below. As shown in this table, the mean angle of the head and tail clusters is always within the angle interval of the assumed third category of lines. Therefore, these clusters can be directly merged as the third category of lines. Since there always are more lines in the direction of the road, Cluster 1, which has the largest sample size, can be deemed as the first category of lines. Among the seven kinds of cluster lines counted, the mean angle in some clusters was not calculated because of the overly small number of lines within the cluster. At present, the number of lines counted has reached a very high proportion. Therefore, except for the head and tail clusters, selecting the lines in the first five clusters can fully realize the screening of the first and second categories of lines.

**FIGURE 7:**
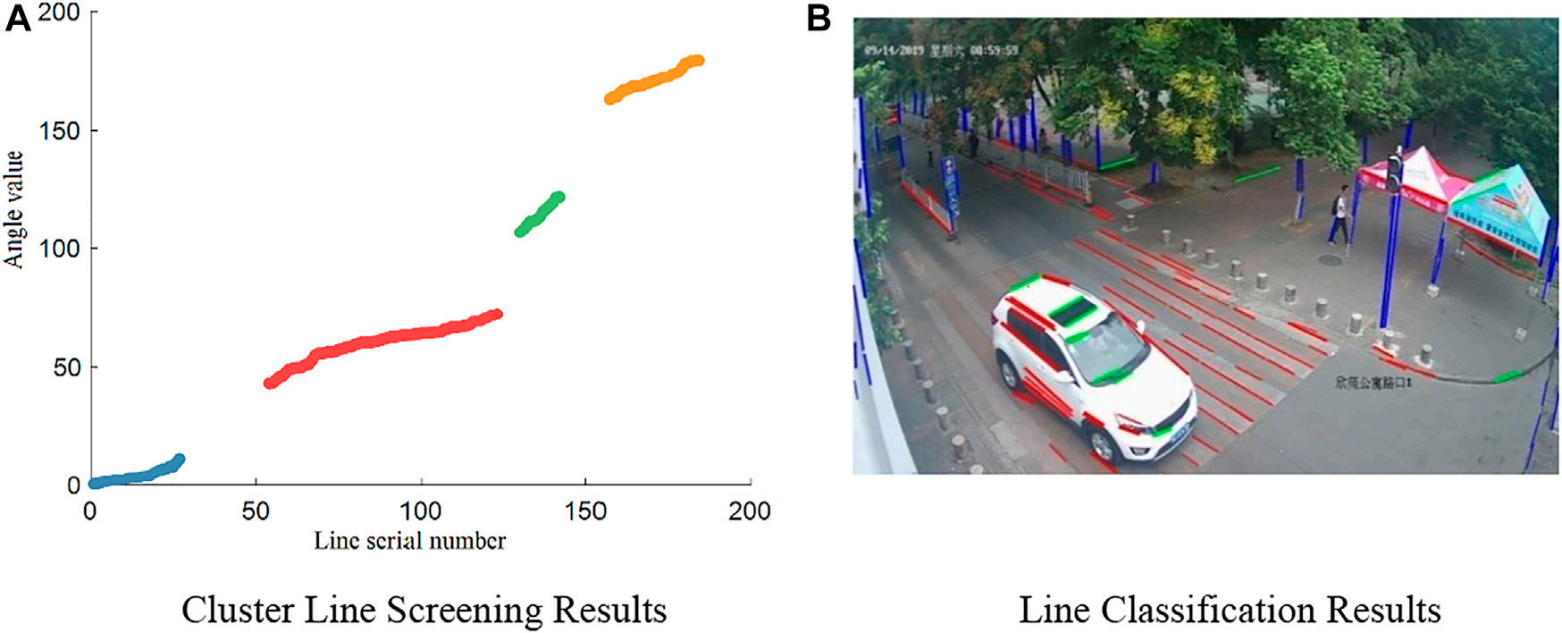
Screening and classification results of cluster lines. **(A)** Cluster line screening results. **(B)** Line classification results.

**FIGURE 8:**
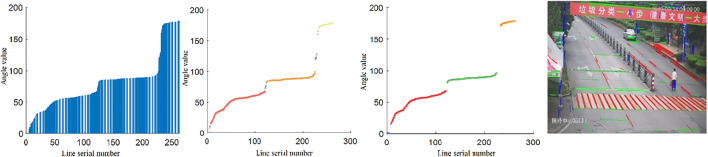
Line screening process.

Under ideal conditions, the images are mainly composed of three categories of lines. The situation at Checkpoint 3 occurs after hierarchical clustering. The four clusters made of the three categories of lines have accounted for 96.07% of the total number of lines. However, the angular relationship between the first and second line categories can be used in other cases. The first line category is always perpendicular to the second in reality. However, because of the intrinsic factors of the camera and other factors related to projection imaging, the angle difference between the two line categories in the image will change to some extent. Nevertheless, compared with other nontarget cluster lines, the angle difference is closest to 90°. For instance, Cluster 4 could be selected as the second line category using the angular relationship in Checkpoint 1. The resulting four clusters selected by considering Cluster 1 as well as the head and tail clusters are shown in Figure 7A. The three categories of lines belonging to the four kinds of clusters are shown in the original image. Figure 7B shows the results. Different colors in the figure represent different categories of lines. Similarly, the screening process for the lines in the images at Checkpoint 2 is also shown in Figure 8.

**TABLE 4:** Mean angle and proportion of the number of lines in different clusters.

Image	Cluster 1	Cluster 2	Cluster 3	Cluster 4	Cluster 5	Head cluster	Tail cluster	Proportion of number (%)
Checkpoint 1	61.66°	37.52°	124.43°	144.76°	86.51°	6.18°	172.02°	94.39
Checkpoint 2	57.94°	114.49°	171.03°	91.50°	—	9.24°	171.84°	94.74
Checkpoint 3	50.13°	88.06°	—	—	—	0.61°	176.23°	96.07
Checkpoint 4	132.68°	161.85°	84.17°	92.60°	102.40°	4.96°	175°	97.39

### Solving the Vanishing Points

The detection and classification of three categories of lines have been completed in the preliminary work. The “line → line” transform (Dubska et al., 2011) in the CHT based on parallel coordinates is used herein to map the lines in the Cartesian coordinate system in an infinite space in the form of broken lines into a finite rhombic space. One of the transformation formulas is as follows. SS represents the CHT of the lines in the first quadrant. (*a, b, c*) was used to represent line *l:ax + by + c = 0*. *d* and *D* represent the distance between two parallel axes in the parallel coordinates in two transformations, respectively.

$$SS_{dD}^{l}\left(\left(a,b,c\right)\right)=\left(db+c,Da+Db,-dDb\right)$$ (5)

To facilitate the subsequent calculation, the image coordinate system was standardized by converting the image coordinate system (*u-v*) with the origin in the top left corner to a standardized rectangular coordinate system (*Cartesian*) with the origin in the middle of the image and the interval range of [−1, 1]. The standardization formula for the coordinate system is as follows. *h* and *w* represent the height and width of the image, respectively.

$$x=\frac{2\times u-w}{w},y=\frac{2\times v-h}{h}$$ (6)

**FIGURE 9:**
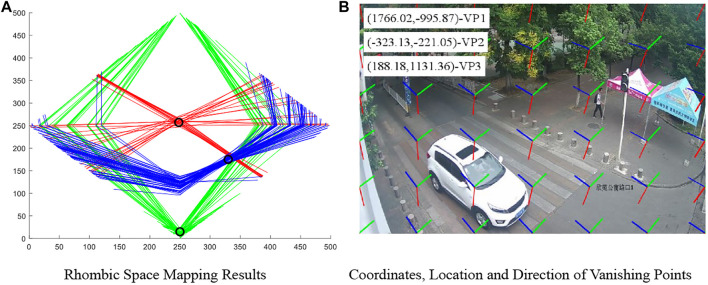
Mapping and coordinate results of rhombic space. **(A)** Rhombic space mapping results. **(B)** Coordinates, location, and direction of vanishing points.

The expression of all lines was solved based on this coordinate system. CHT was conducted on the lines obtained, and the lines were mapped to the rhombic space to obtain the corresponding broken lines. Figure 9A shows the mapping of the three categories of lines in the image to the rhombic space. The three categories of lines are shown in red, green, and blue, respectively. The corresponding lines generate obvious convergence (the circle in the figure) after the mapping into the rhombic space. The coordinates of vanishing points can be obtained by finding the maximum weight after rasterizing the whole rhombic space. Since the minor difference in the angle between lines affects the intersection projected onto the rhombic space, the whole rhombic space is searched by small areas when voting for the maximum weight. Generally, the small areas are in the size of 3 × 3. For the target small areas finally obtained, the central point is recorded as the vanishing point.

After obtaining the coordinates of vanishing points in the rhombic space, the vanishing point coordinate in the standardized rectangular coordinate system can be obtained using the following formula. The subscript *o* represents the original Cartesian coordinate system, and the subscript *d* means the coordinate system in the rhombic space. Finally, the coordinates of vanishing points were destandardized to restore to the original image coordinate system. That is, Formula 6 was used reversely. The final results of vanishing point detection are shown in Figure 9B. VP1, VP2, and VP3 represent three kinds of vanishing points, respectively. The short lines in different colors represent different locations and directions of different vanishing points.

$${\left[x,y,w\right]}_{d}\to {\left[Dy,sgn\left(x\right)dx+sgn\left(y\right)Dy-dDw,x\right]}_{o}$$ (7)

## Experimental Results and Analysis

### Comparative Analysis of Vanishing Point Detection Methods

The experimental platform for the method herein is MATLAB 2018a, and the resources for the computing power were allocated using a PCI5-9400 CPU@2.90 GHz Processor in a 64-bit operating system. To evaluate the performance of the method herein for single vanishing point detection, the experiment was conducted on DARPA (Moghadam et al., 2012), which is a data set composed of 501 natural images with a size of 320 × 240. In each image, there are manually labeled vanishing point coordinates as vanishing point truth values. The error of vanishing point detection is measured by calculating the normalized *x*-axis distance, normalized *y*-axis distance, and normalized Euclidean distance between the vanishing points detected and the vanishing point truth values (Yu and Zhu, 2019).

The normalized *x*-axis distance is defined as follows:

$$NormDistx=\frac{\left|x-x_{0}\right|}{w}$$ (8)

The normalized *y*-axis distance is defined as follows:

$$NormDisty=\frac{\left|y-y_{0}\right|}{h}$$ (9)

**FIGURE 10:**
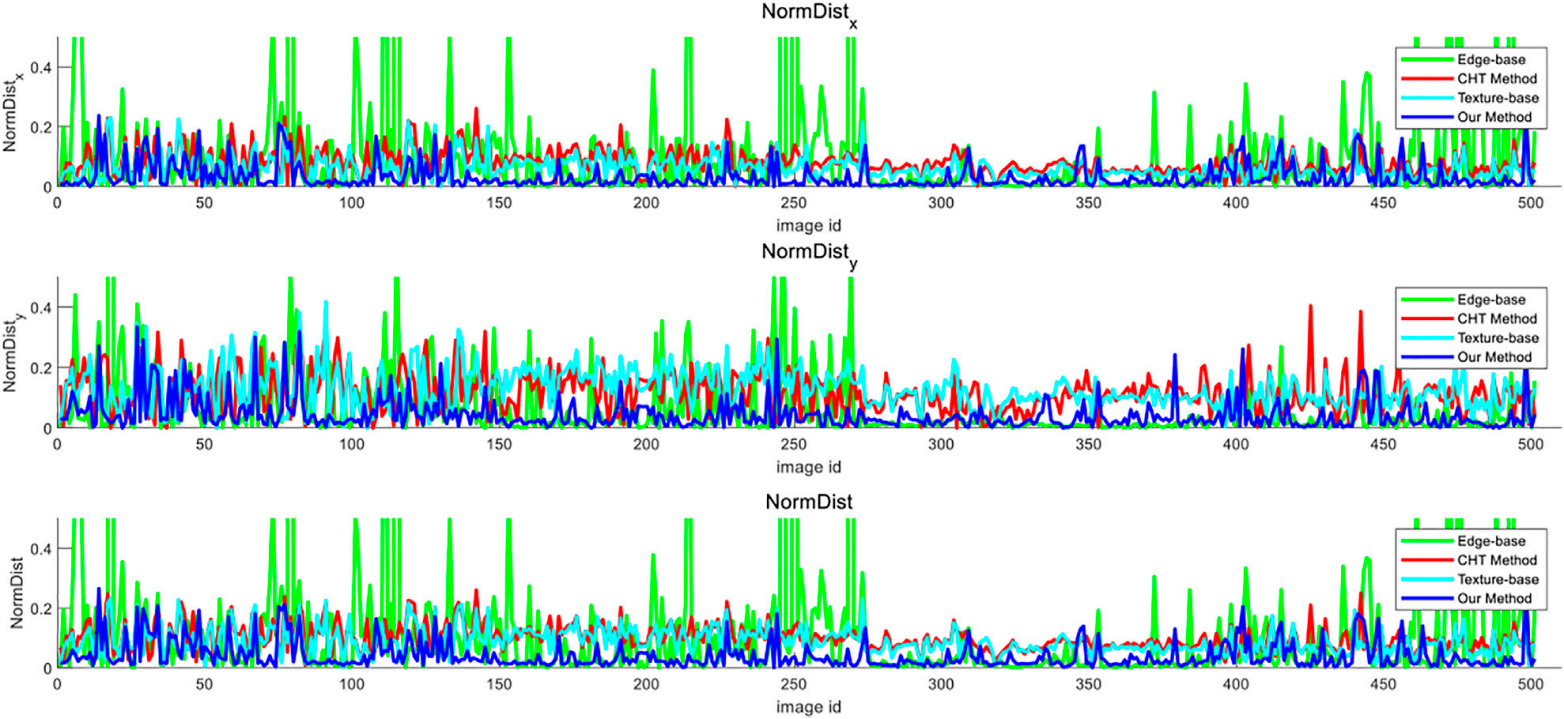
Comparison of error curves of different vanishing point detection methods.

The normalized Euclidean distance is defined as follows:

$$NormDist=\frac{\sqrt{{\left(x-x_{0}\right)}^{2}+{\left(y-y_{0}\right)}^{2}}}{\sqrt{w^{2}+h^{2}}}$$ (10)

where (*x, y*) and (*x*_0_ *, y*_0_) are the coordinates of the vanishing points detected and the vanishing point truth values, respectively, and *w* and *h* are the width and length of images, respectively. It can be seen from the formulas that the smaller the value obtained, the higher the accuracy of the vanishing points detected. The method was tested based on CHT (Markéta and Herout, 2013), the texture-based method (Bui et al., 2014), the edge-based method (She et al., 2015), and the method proposed herein on the data set. The comparison of the vanishing point detection error among the four methods is shown in Figure 10. It can be seen that the protrusion of the error curve of the edge-based method is prominent, whereas the curve of the method herein has smaller fluctuations than other algorithms. Therefore, the method herein is more accurate in detecting a single vanishing point.

**FIGURE 11:**
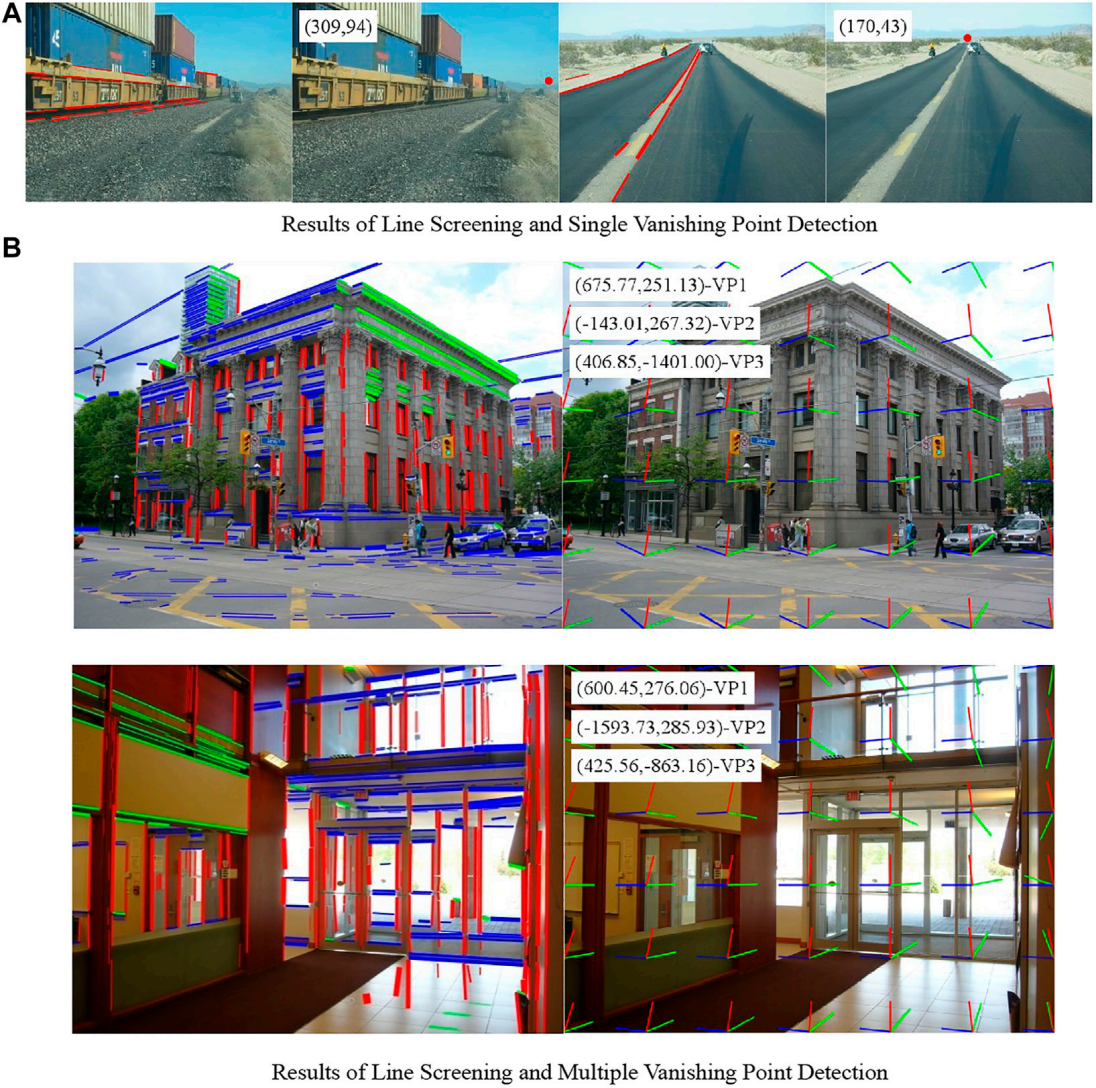
Results of line screening and vanishing point detection. **(A)** Results of line screening and single vanishing point detection. **(B)** Results of line screening and multiple vanishing point detection.

For further comparison, the mean of the three normalized distances of different methods and the speed of vanishing point detection are calculated, as shown in Tables 5, 6. According to the tables, the average error of the method herein is the smallest on the normalized Euclidean distance, so it performs better on single vanishing point detection. The method herein also runs for a shorter time than other methods and can meet the requirements for timeliness. The results of vanishing point detection in some images in the data set, including the screening of lines, are shown in Figure 11.

**TABLE 5:** Comparison of average error.

	Edge-base	CHTMthod	Texture-base	OurMethod
NormDist_x_	0.0976	0.0789	0.0612	0.0523
NormDist_y_	0.1518	0.1146	0.1262	0.1439
NormDist	0.1247	0.0994	0.0878	0.0711

**TABLE 6:** Comparison of running speed.

	Edge-base	CHTMthod	Texture-base	OurMethod
Time(s)	0.3862	0.2501	7.3606	0.4094

### Analysis of Multiple Vanishing Point Detection Performance

Although the performance of the method herein for single vanishing point detection is explained in the previous section, the main purpose of this paper is to detect the vanishing points in three directions at checkpoints. Therefore, the detection of vanishing points in three directions was further experimented on the York Urban Database. The database is composed of 102 urban environment images with a size of 640 × 480, mainly scenes from the campus of York University and downtown Toronto, Canada. Moreover, the database satisfies the Manhattan hypothesis. There are three orthogonal vanishing point truth values in each image. Therefore, the database can be used to evaluate the vanishing point detection performance in three directions.

**FIGURE 12:**
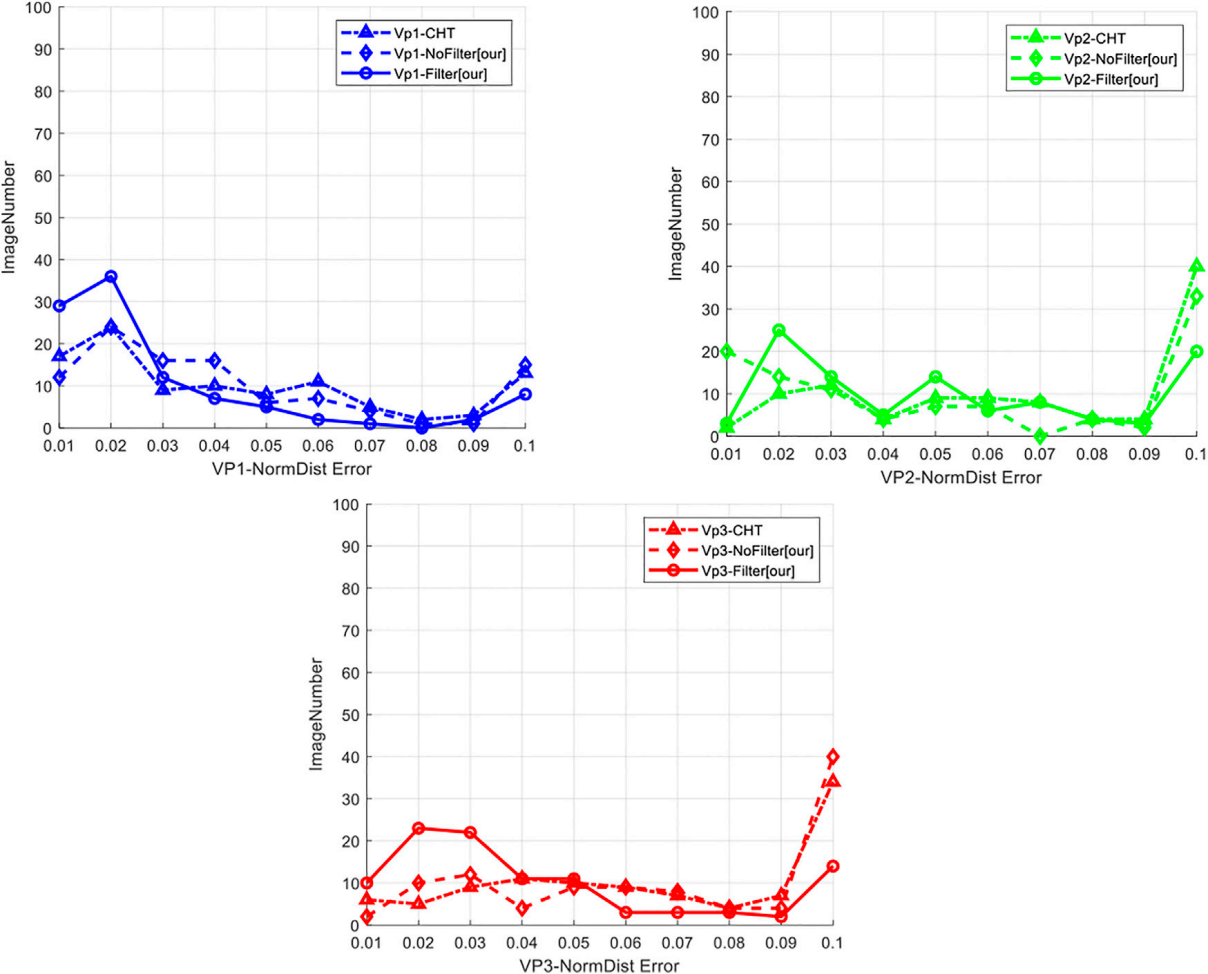
Error distribution diagram for three kinds of vanishing points.

Split testing was performed on the method based on CHT (Markéta and Herout, 2013) and the method herein (using and not using line filtering) on this database to calculate the normalized Euclidean distances of the three vanishing points, respectively. The number of images with the NormDist value falling into different intervals [range (0,0.1) and step size 0.01] was counted. All images with NormDist >0.1 were included in NormDist = 0.1, obtaining the error distribution of vanishing point detection as shown in Figure 12.

It can be seen from the error distribution diagram for three kinds of vanishing points that the method herein, in which line filtering was used, was slightly leading in the accuracy of the detection of the three kinds of vanishing points, whereas the performance of the method without using line filtering was close to the method (Markéta and Herout, 2013). The mean of the normalized Euclidean distance was also calculated. The results are shown in the following Table 7. The accuracy of this method was low when line filtering was not used. When line filtering was performed, the accuracy was significantly improved, especially in detecting the second and third kinds of vanishing points. This also indicates that the accuracy of vanishing point detection can be improved by filtering short lines. The results of vanishing point detection in some images in the database, including the screening of lines, are also shown in Figure 11B.

**TABLE 7:** Comparison of average error.

	VP1- NormDist	VP2- NormDist	VP3- NormDist
CHT	0.0575	0.0942	0.0545
Our-Nofilter	0.0561	0.0925	0.0559
Our-filter	0.0557	0.085	0.0466

## Conclusion

This paper mainly realized the detection of vanishing points on roads under surveillance cameras at checkpoints on urban roads. LSD algorithm was used to detect the line segments in the image and filter out short lines using the relation between the line length and the pixel. In addition, line segments were screened and classified by hierarchical clustering and in combination with the image features and the similarity change rule of the angle of line segments on this basis to select the main line segments and eliminate some interferential line segment data. The classified three categories of lines were mapped into a rhombic space, respectively, for the relevant calculation to obtain three vanishing points. The three vanishing point directions can be used to construct the three-dimensional coordinate system in the image, which facilitates the subsequent processing of the image and other computer vision tasks. The method in this paper can reduce the amount of line segment data to be processed to some extent, improve the running speed and eliminate the interference of a large amount of irrelevant data, and greatly improve the accuracy. Therefore, it is of certain theoretical significance and practical value.

## Data Availability

The raw data supporting the conclusions of this article will be made available by the authors, without undue reservation.
